# Long-Term Clinical, Audiological, Visual, Neurocognitive and Behavioral Outcome in Children With Symptomatic and Asymptomatic Congenital Cytomegalovirus Infection Treated With Valganciclovir

**DOI:** 10.3389/fmed.2020.00268

**Published:** 2020-07-24

**Authors:** Arianna Turriziani Colonna, Danilo Buonsenso, Davide Pata, Gilda Salerno, Daniela P. R. Chieffo, Domenico M. Romeo, Valerio Faccia, Guido Conti, Fernando Molle, Antonio Baldascino, Chiara De Waure, Anna Acampora, Rita Luciano, Rosaria Santangelo, Piero Valentini

**Affiliations:** ^1^Istituto di Pediatria, Università Cattolica del Sacro Cuore, Rome, Italy; ^2^Department of Woman and Child Health and Public Health, Fondazione Policlinico Universitario A. Gemelli IRCCS, Rome, Italy; ^3^Istituto di Microbiologia, Università Cattolica del Sacro Cuore, Rome, Italy; ^4^Istituto di Neuropsichiatria, Università Cattolica del Sacro Cuore, Rome, Italy; ^5^Unit of Pediatrics, Department of Gynecologic, Pediatric and Neonatologic Sciences, “Sant'Andrea” University Hospital, University “Sapienza” of Rome, Rome, Italy; ^6^Department of Aging, Neurologic, Orthopedic and Head and Neck Science, Fondazione Policlinico Universitario A. Gemelli, IRCCS, Rome, Italy; ^7^Istituto di Otorinolaringoiatria, Università Cattolica del Sacro Cuore, Rome, Italy; ^8^Istituto di Oculistica, Università Cattolica del Sacro Cuore, Rome, Italy; ^9^Department of Experimental Medicine, University of Perugia, Perugia, Italy; ^10^Istituto di Igiene, Università Cattolica del Sacro Cuore, Rome, Italy; ^11^Department of Microbiology and Infectious Diseases, Fondazione Policlinico Universitario A. Gemelli IRCCS, Rome, Italy

**Keywords:** congenital cytomegalovirus, valganciclovir, hearing loss, congenital infections, neurocognitive outcome

## Abstract

Cytomegalovirus (CMV) is the most common cause of congenital infection in humans. There are no enough data on long-term outcome of newborns with congenital CMV (cCMV) infection, particularly for those asymptomatic at birth. For this reason, we performed this study to evaluate long-term audiological, visual, neurocognitive, and behavioral outcome in patients with symptomatic and asymptomatic cCMV infection treated with oral Valganciclovir (VGC). Thirty-six newborns with confirmed cCMV infection were evaluated: 12 (33.3%) symptomatic at birth and 24 asymptomatic (66.7%). No one had cognitive impairment. Cognitive assessment scales resulted abnormal in 4/35 patients (11.4%). 11/21 patients (52.4%) achieved abnormal scores in neuropsychological tests. The language evaluation gave pathological results in 6/21 (28.5%) patients. 6/35 patients (17.1%) developed SNHL, all symptomatic at birth except one. None of the 34 patients evaluated developed CMV retinopathy. Our study shows that both symptomatic and asymptomatic newborns with cCMV infection develop long-term sequelae, particularly in the behavioral and communicative areas, independently from the trimester of maternal infection.

## Introduction

Cytomegalovirus (CMV) is the most common cause of congenital infection in humans (1) and the leading cause of cognitive impairment and non-genetic sensorineural hearing loss (SNHL) in infancy (2). Congenital CMV (cCMV) infection is symptomatic in about 10–15% cases with a perinatal mortality of 10%; 90% of those who survive develop neurological sequelae, mainly defects of psychomotor development and SNHL (3). Eighty five to ninety percent of newborns does not present any symptoms at birth; however, 8–15% of these will show late signs related to cCMV, especially SNHL (4–8). Moreover, European and Asian studies have shown how cCMV infection can have repercussions on multiple developmental areas (9, 10), although the long-term outcome of newborns with asymptomatic infection is not well clear. Kimberlin et al. (11) demonstrated that intravenous (iv) Ganciclovir (GCV) for 6 weeks in symptomatic cCMV with CNS involvement prevents deterioration of the auditory and psychomotor function. Furthermore, antiviral therapy improved the neurological outcome during follow-up (12). However, this therapy requires prolonged hospitalization and vascular catheters with increased risk of nosocomial infections. Valganciclovir (VGC) is the pro drug of GCV (GCV L-valil-ester); first studies showed that a VGC dose of 15 mg/kg orally every 12 h is comparable to 6 mg/kg every 12 h iv of GCV (13). Several studies evaluated the effectiveness of oral VGC, but all of them aimed at symptomatic cCMV (14). Due to the lack of clear data on long-term follow-up of cCMV infection, we performed this study aiming to evaluate long-term, clinical, audiological, visual, neurocognitive, and behavioral outcome in patients with symptomatic and asymptomatic cCMV infection treated with VGC.

## Materials and Methods

We performed a retrospective study of patients with cCMV infection (both symptomatic and asymptomatic) treated with VGC, evaluated from October 2009 to February 2017. The study was approved by the Ethical Committee of our institution (prot 26317/19 ID 2629).

### Maternal Diagnosis

Mothers have been considered infected in presence of at least one of the following:

- seroconversion with appearance of anti-CMV IgG antibodies documented during pregnancy,
- in case of positive anti-CMV IgG at the first serological control in pregnancy, if the IgG avidity index was compatible with an infection acquired after conception,
- presence of CMV-DNA in blood and urine (15).

Women with IgG positive antibodies before pregnancy and those with high IgG Avidity without IgM during the first 25 weeks of gestation were classified as having non-primary CMV infection.

### Neonatal Diagnosis

Infants were considered infected if CMV-DNA was found in blood or urine using the Real TimePolymerase Chain Reaction method (RT-PCR), no later than the first 3 weeks of extra-uterine life (11, 16). It is a commercial assay and the analytical sensitivity allowed the quantification of 200 to 10^6^molecules of the target DNA.

All infected newborns underwent clinical evaluation (17), blood tests, assessment of the ocular fundus, audiological screening using otoemissions (TEOAEs), auditory brainstem response (ABR), ultrasound of the brain and, in doubtful cases, encephalic magnetic resonance, and cranial radiography.

Newborns were classified as symptomatic if they had at least one of the following findings (18): petechiae, hepatomegaly, splenomegaly, abnormalities in blood chemistry (thrombocytopenia <100,000/μl, anemia, leukopenia, elevation of liver enzymes, conjugated hyperbilirubinemia), SGA < -2 DS status, neurologic and/or ophthalmologic examination anomalies, microcephaly, convulsions, neuroradiological abnormalities related to CMV infection, abnormalities in the ABR exam. Ultrasonographic signs indicative of symptoms included calcifications, cystic periventricular leukomalacia, subependymal pseudocysts, germinolytic cysts, white matter anomalies, cortical atrophy, migration disorders, cerebellar hypoplasia, and lenticulo-striatal vasculopathy (the latter only if in association with other signs) (19).

### Treatment

Patients were treated with a galenic preparation of oral VGC based on data available in literature (13, 14, 20–22). The galenic was set up according to the dictates of rules of good preparation (N.B.P.) indicated on the Official Pharmacopoeia XII ed. following the procedure reported in the literature (23).

The treatment was started in the first month of age. Patients received VGC at a dose of 32 mg/kg/day divided into two daily doses, for a variable number of 6-week cycles (up to the persistently negative viremia, as stated below). At the beginning, on day 21 and at the end of the 6-week therapy cycle, the following parameters were monitored: viral load by RT-PCR performed on whole blood, urine and pharyngeal swab; creatinine, SGPT, amylase, gamma-GT, alkaline phosphatase and blood-cell count with formula.

At the end of each cycle, monthly and until the first year of age, patients underwent clinical evaluation and determination of viral load on blood and urine; if viremia was found positive again, a new 6-week therapy cycle was started, with the same modalities.

Patients born after the publication of Kimberlin's study in 2015 received the drug for 6 months, in accordance with the evidence that emerged from the study (14).

The administration of the drug was suspended in 1 case with peripheral blood neutrophil count lower than 500 cells/μl (reversible side effect).

### Follow-Up

Patients with confirmed cCMV infection underwent audiological, neurocognitive, psychological, ocular, audiological, and neurological assessments; tests performed are summarized in Table 1.

**Table 1:** Tests performed for the evaluation of outcomes of primary and secondary interest, categories analyzed, and sample or sub-sample in which they are performed.

**Outcome**	**Analyzed categories**	**Sample or subsample**
**NEUROCOGNITIVE OUTCOME**		
Test 1: WPPSI-III: Wechsler Preschool and Primary Scale of Intelligence—III	IQ ≤ 69: score lower than normal; 70 ≤ IQ ≤ 84: borderline score; 85 ≤ IQ ≤ 115: normal score; IQ ≥ 116: higher than the norm score.	Patients from 2.6 to 7.3 years
Test 2: WISC-IV: Wechsler Intelligence Scale for Patients-IV	IQ ≤ 69: score lower than normal; 70 ≤ IQ ≤ 84: borderline score; 85 ≤ IQ ≤ 115: normal score; IQ ≥ 116: higher than the norm score.	Patients from 6 + 0 to 16 + 11 years
Test 3: Leiter-R: non-verbal scale	IQ ≤ 69: score lower than normal; 70 ≤ IQ ≤ 84: borderline score; 85 ≤ IQ ≤ 115: normal score; IQ ≥ 116: higher than the norm score.	Patients from 2 to 20 years of foreign language
**NEUROPSYCHOLOGICAL OUTCOME**		
Test 1: NEPSY-II	Scores from 1 to 4: deficit; from 5 to 7: lower than the norm; from 8 to 12: in the norm; from 13 upwards: above the norm.	Patients from 3 to 16 years
Test 2: Bells test	Result >-1.66 DS: normal; < -1.66: lower than the norm	Patients from 4 to 8 years
**LANGUAGE**		
Test 1: BVL_4–12: Battery for the evalaution of language in patients from 4 to 12 years	Result < -1.5 DS: lower than the norm; >-1.5 DS: normal	Patients from 4 to 12 years
Test 2: Phonolexical Test (TFL)	≤ 50° percentile: lower than normal; >50° percentile: normal; >90°: higher than normal.	Patients from 3 to 6 years
Test 3: Griffiths battery – C scale in five patients (comprehension and verbal production scale)	Score ≤ 69: score lower than normal; 70 ≤ score ≤ 84: borderline score; 85 ≤ score ≤ 115: score in the standard; score ≥ 116: score higher than normal.	Patients up to 3 years
**BEHAVIOR**		
Test 1: Child Behavior Checklist 1½−5 (CBCL)	>60: normal; =60: borderline; >60: lower than the norm.	Patients from 1 to 5 years
Test 2: Child Behavior Checklist 6–18 (CBCL)	>60: normal; =60: borderline; >60: lower than the norm.	Patients from 6 years upwards
**RETINOPATHY**		
Test 1: Fundus oculi examination	Present/absent retinopathy	All the patients
**HEARING OUTCOME**		
Test 1: TEOAEs	Pass (normal)/ Refer (pathological)	All patients at the 3rd day of life
Test 2: ABR Auditory threshold	Normoacusia if ≤ 20 dB Unilateral or bilateral hypoacusia: mild 21–40 dB; average 41–70 dB; severe 71–90 dB; deep > 90 dB.	0–2/3 years of age depending on the collaboration
Test 3: Audiometry Auditory threshold	Normal if ≤ 20 dB Unilateral or bilateral hypoacusia: mild 21–40 dB; average 41–70 dB; severe 71–90 dB; deep > 90 dB.	From 2 to 3 years of age upwards

### Primary Outcome

Evaluation of long-term clinical, audiological, visual, neurocognitive, and behavioral outcome in patients with symptomatic and asymptomatic cCMV infection treated with VGC.

### Secondary Outcome

Association between outcome (clinical, audiological, visual, neurocognitive, and behavioral) and viremia, number of treatments performed and trimester of maternal infection.

### Statistical Analyses

The analysis of data includes a descriptive part of the sample carried out by constructing frequency tables (absolute and percentages) for the categorical variables and with the mean ± standard deviation for the quantitative variables.

The association between the dependent and independent variables has been tested using statistical tests defined on the basis of the nature of the analyzed variables. For the analysis of the association between the symptomatic or asymptomatic condition at birth and the neurocognitive, neuropsychological, language, behavioral, auditory, and long-term retinopathy outcomes, a univariate analysis has been performed using the Chi-square test and the Fisher's exact test. These outcome variables have been categorized based on the score obtained in the tests and investigations carried out, previously described and reported in Table 1; the same tests have been used to study the association between the trimester of pregnancy in which the CMV infection occurred and the outcomes.

The Mann–Whitney test was used to analyze the endpoints regarding the number of therapy cycles administered.

For all analyses, a *p* < 0.05 was considered significant.

The analyses were performed using the STATA software version 13.1.

## Results

### Study Population

Thirty-six newborns with confirmed cCMV infection: 12 symptomatic patients at birth (33.3%) and 24 asymptomatic (66.7%), who underwent oral VGC treatment were included in the study. The average age of the follow up is 4.23 years ± 1.57 SD. All patients were in good general health conditions, no endocrinological disorders were diagnosed.

Tables 2–4 summarize the characteristics of population, the treatment cycles, and the comparison between symptomatics and asymptomatics, respectively.

**Table 2:** Patient characteristics.

**Patient**	**Gestational Age**	**Birth Weight (g)**	AGA/SGA/LGA *	**Symptomatic (S)/Asymptomatic (A)**	**CMV PCR in urine, blood, pharynx**	**Viral load in blood (copies/mL)**	**Hearing status**	**Thrombocytopenia**	**Petechiae**	**Hepatomegaly**	**Splenomegaly**	**CNS involvement**	**Hepatitis**	**Trimester of infection**
#1	40	3,470	AGA	A	+ U, B, P	930								2
#2	39	3,140	AGA	A	+ U, B, P	6,760								2
#3	40	2,660	SGA	A	+ U, B, P	17,190								2
#4	38	3,250	AGA	S	+ U, B, P	9,340	Left SNHL					White matter abnormalities		2
#5	40	2,830	AGA	S	+ U, B, P	152,800						White matter abnormalities, cysts, scars		1
#6	38	3,330	AGA	A	+ U, B, P	5,604								1
#7	40	3,700	AGA	A	+ U, B, P	1,880								3
#8	37	2,600	AGA	A	+ U, B, P	431								3
#9	38	3,240	AGA	A	+ U, B, P	486								2
#10	38	2,750	AGA	A	+ U, B, P	1,820								2
#11	37	3,250	AGA	A	+ U, B, P	2,000								1
#12	39	3,380	AGA	A	+ U, B, P	17,640								1
#13	40	3,930	LGA	A	+ U, B	#								1
#14	40	3,450	AGA	A	+ U, B, P	9,600								2
#15	38	2,740	SGA	S	+ U, B, P	9,200	Left SNHL							2
#16	40	4,220	LGA	A	+ U, B, P	810								3
#17	37	3,500	LGA	S	+ U, B, P	304						White matter abnormalities		3
#18	39	3,120	AGA	A	+ U, B, P	5,856		X	X	X		Subependymal pseudocysts		1
#19	39	2,920	SGA	S	+ U, B, P	8,684								3
#20	38	3,150	AGA	A	+ U, B, P	3,753						Lenticulostriatal vasculopathy		1
#21	#	#	#	A	+ U, B, P	1,987								3
#22	41	3,200	AGA	A	+ P	0								2
#23	#	#	#	A	+ U, P	0								1
#24	40	3,340	AGA	S	+ U, B, P	31,604	Left SNHL					Subependymal pseudocysts		1
#25	40	2,800	SGA	A	+ U, B, P	31,900	Left SNHL							1
#26	40	3,720	AGA	S	+ U, B, P	519						White matter abnormalities, lenticulostriatal vasculopathy		2
#27	#	#	#	S	+ U, B, P	3,610						Hypotonia, seizures		#
#28	39	4,000	LGA	A	+ U, B, P	#								3
#29	36	2,730	AGA	A	+ U, B, P	2,774								2
#30	35	1,750	<3	S	+ U, B, P and cerebrospinal fluid	1,746,873	Bilateral SNHL	X						1
#31	38	3,670	LGA	A	+ U, B, P	9,541								3
#32	38	3,130	AGA	S	+ U, B, P	3,883						White matter abnormalities, subependymal pseudocysts		1
#33	38	2,570	SGA	A	+ U, B, P	2,343								1
#34	38	3,580	AGA	S	+ U, B, P	#						Subependymal pseudocysts, lenticulostriatal vasculopathy		3
#35	37	4,180	LGA	S	+ U, B, P	774	Bilateral SNHL					White matter abnormalities, lenticulostriatal vasculopathy		1
#36	38	4,150	LGA	A	+ U, B, P	#								1

#*missing data*.

**Table 3:** Treatment cycles.

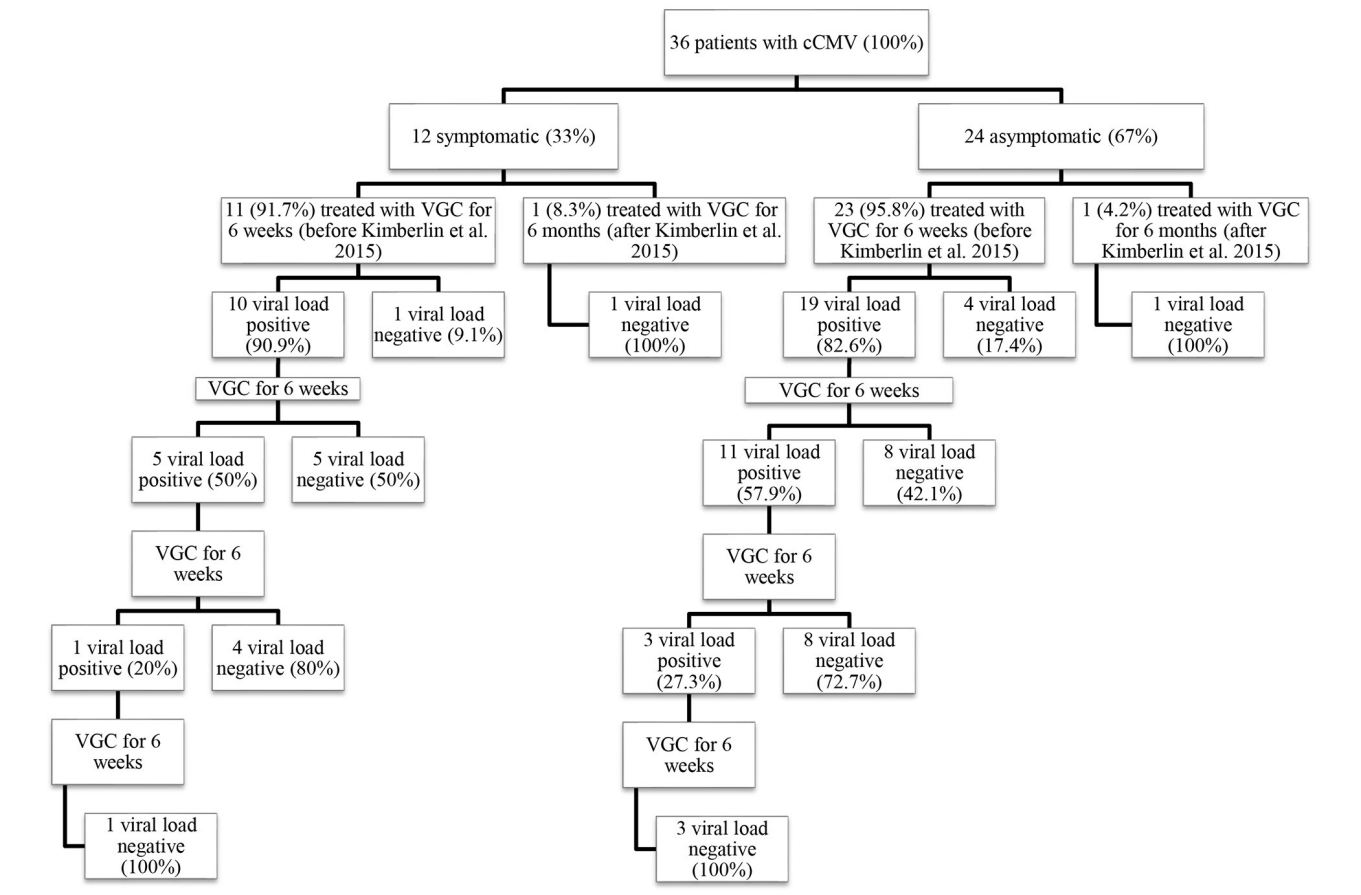

**Table 4:** Comparison between Symptomatics and Asymptomatics.

	**Gestational age (median)**	**Birth Weight (g, median)**	**Time of maternal infection (median trimester)**	**Viral load in blood (copies/mL)**	**Cycles of VGC (*n*)**	**SNHL (*n*)**	**Retinopathy (*n*)**	**Cognitive Impairment (*n*)**	**Abnormal neuropsycological tests (*n*)**	**Language disorders (*n*)**	**Abnormal behavioral tests (*n*)**
Symptomatics	38.18	3,176	1.81	178,872	2.33	5	0	1	2	3	2
Asymptomatics	38.77	3,296	1.83	6,490	2.38	1	0	2	9	3	6

Based on the ESPID criteria (18), of the 12 symptomatic newborns, eight (66.5%) were severe symptomatic at birth, 1 (8%) moderate symptomatic and 1 (8%) mild symptomatic. Two patients (17%) were born with unilateral hearing loss, in association with subependymal pseudocysts in a newborn and isolated in another.

Timing of maternal infection was available for 35 patients (Table 5):

- first trimester for 15 women (41.7%), giving birth to 6 symptomatic newborns (40%);
- second trimester for 11 women (30.6%); at birth 3 newborns (27%) were symptomatic;
- third trimester for 9 women (25%), giving birth to 2 symptomatic newborns (22%) with CNS involvement.

**Table 5:** Study population according to trimester of maternal infection and symptoms at birth.

**Gestational age and symptoms at birth**			
	**Symptomatic**	**Asymptomatic**	**Total (*****n=36*****)**
**First trimester**	6 (16.7%)	9 (25%)	15 (41.7%)
**Second trimester**	3 (8.3%)	8 (22.2%)	11 (30.6%)
**Third trimester**	2 (5.6%)	7 (19.4%)	9 (25%)
**Nondescript**	1 (2.8%)		1 (2.8%)
**Total**	12 (33.3%)	24 (66.7%)	36

### Results of Neurocognitive and Behavioral Follow-Up

Neuropsychiatric evaluation was proposed to 35 patients, four of whom did not complete the tests. However, not all continued the follow-up in the following years and consequently performed the different tests, unlike the audiological follow-up. With regard to cognitive development, 30 out of 33 evaluated patients (90.9%) were normal (Intelligence Quotient, IQ, ≥85). The average IQ of patients in the group is 110.9. Specifically, 15 patients have a development above the norm with IQ ≥ 116 (45.5%) and 15 in the standard with 85 ≤ IQ ≤ 115 (45.5%). Among them, eight patients scored below the norm in at least one subtest. The major recurrence was observed in the test called Cifrario (10 patients out of 22–45.5%). Three patients achieved a borderline score: 70 ≤ IQ ≤ 84 (9.1%), one was severely symptomatic at birth. No one had cognitive impairment (IQ ≤ 69). The Symbols' Search (SS) test of cognitive assessment scales, given to 22 patients, was abnormal in 4 patients in whom the viremia for CMV had not become undetectable after the first course of therapy (*p* = 0.017, Table 6).

**Table 6:** Statistic analysis of the Symbols' Search subtest results (neurocognitive examination) and the zeroing of CMV viremia after 1 cycle of oral VGC (*n* = 22).

			**CMV viremia after 1 cycle of oral VGC**		
			**Positive**	**Negative**	***p-*value**
Neurocognitive subtest (Symbols' Search)	Normal	No. of patients	5	13	0.017
		%	55.6%	100.0%	
	Lower than normal	No. of patients	4	0	
		%	44.4%	0.0%	

Of the 21 patients who underwent neuropsychological tests, 11 (52.4%) achieved an insufficient score in at least one subtest with a more frequent fall in the attention tests (7 out of 19−36.8%) and semantic fluency (8 out of 12−66.7%).

The language evaluation carried out on 32 patients gave normal results in all but in 6 patients, including 3 symptomatic at birth with CNS involvement; a significant difference emerged between those with language disorders and without in relation to the number of cycles of VGC administered. In particular, the median number of cycles is 3.50 with IQR = 2 in the first group and 2 with IQR = 1 in second group (*p* = 0.042 in the Mann–Whitney test).

Twenty-eight families completed the CBCL questionnaire for the analysis of the child's behavior. In the Total Scale of Problems, four patients (14.3%) obtained pathological results on the Internalizing scale and two patients (7.1%) also in the Externalizing scale. The same two patients were symptomatic at birth with CNS involvement.

Seven patients in total obtained an alarming score on the Internalizing scale (25%), three of them had a pathological score on the Externalizing scale and four of them were pathological on the Total scale.

The score on the Externalizing scale was noteworthy for four patients (14.3%), two of them already reported in the Total scale, and three also with internalizing problems.

Importantly, abnormal neurocognitive and behavioral tests were obtained both in newborns infected in the first trimester (9/15, 60%) and in the third trimester (4/9, 44%), *p* > 0.05.

### Results of the Audiological Follow-Up

Thirty-five patients underwent audiological follow-up. It was assessed by Auditory Brainstem Response (ABR) within the 3rd month of age. The repetition of the exam was proposed at 6, 12, and 18 months of age and then annually until school age. The average age of the follow up is 4.23 years ± 1.57 SD.

Six patients (17.1%) developed SNHL, all symptomatic at birth except one (*p* = 0.012—Table 7). One child has had unilateral left-sided deep hearing loss (auditory threshold 95 dB−5 years of age at the last instrumental control) since the first control. Two patients, both 4 years old, have had severe unilateral left-sided hearing loss (auditory threshold 80 and 85 dB) since the first control, which has remained stable over time.

**Table 7:** Statistic analysis of the association between the presence of hearing loss and the presence of signs and symptoms of congenital CMV infection at birth.

			**Symptoms at birth**		
			**No**	**Yes**	***p-*value**
Hearing impairment	No	No. of patients	22	7	0.012
		%	95.7%	58.3%	
	Yes	No. of patients	1	5	
		%	4.3%	41.7%	

A child with normal ABR at birth, who had not performed controls in the first year of life, resulted affected by bilateral hypoacusia with 50 dB thresholds on the left and 60 dB on the right ear in the 2nd year of age.

In a child, symptomatic at birth, with known bilateral hearing loss since the first control and already wearing a prosthesis in the right ear, a worsening at the age of 3 years was observed (threshold 90 dB on the right and 50 dB on the left ear). He then started using a prosthesis also for the left ear and performed a 6-month course of therapy with VGC after which the hearing thresholds remained stable.

The remaining 29 patients (82.9%), including 7 symptomatic and 22 asymptomatic, had a physiological result at the auditory function control, showing a normal bilateral acoustic threshold (<20 dB) or slightly increased (25–30 dB) because of transmission problems for upper respiratory tract infections, as documented by the contextual clinical evaluation and by the results of the impedance test (tympanograms B or C).

### Results of Ophthalmological Follow-Up

Thirty-four patients (12 symptomatic at birth) underwent examination of the fundus of the eye. No child developed CMV retinopathy.

## Discussion

Our study has analytically explored the long-term neurocognitive, behavioral, auditory, and ophthalmological outcome of a group of symptomatic and asymptomatic patients affected by cCMVand treated with VGC, showing a considerable impact of cCMV infection on social and individual child health.

As regards the cognitive domain, except for the case of a child with severe nervous system involvement since birth, patients achieved excellent results. There was no finding of overt cognitive deficit and only three patients obtained a borderline score (IQ = 71, 77, and 79). Two of these patients were asymptomatic at birth and one was diagnosed with Language and Attention Deficit and Disorder of Language Understanding.

Fifteen patients out of 33 brilliantly faced cognitive tests, reaching scores above the norm. Overall, the average IQ of patients in the group is in the standard. Interestingly, our values are significantly higher than the average IQ of the group of asymptomatic infected, not treated with VGC, studied by Zhang et al. (10): IQ of 89.43 ± 12.78 among 49 patients between 2 and 6 years.

Korndewal's et al. (9) published in 2017 data on a 6-year multidisciplinary follow-up of a group of patients with untreated symptomatic and asymptomatic cCMV. We compared our results with Korndewal study, although there are intrinsic methodological differences between the two projects, such as sample size, classification of symptomatic and asymptomatic at birth, evaluation tests, characteristics of the population, cultures. In our series a better neuropsychiatric outcome emerges, in particular the absence of cognitive deficit vs 6% (3.7% among asymptomatic patients) in the Dutch group. However, our patients still presented specific falls in the SS test of cognitive assessment scales (4 patients). The SS test helps assess the child's processing speed, measures the ability to focus attention, speed of analysis and capacity for discrimination (24). Interestingly, there was an association between lower scores and the lack of negative viremia after the first cycle of VGC (*p* = 0.017 at Fisher's exact test).

On the contrary, the rate of generic language disorder was similar: 18.8% of patients in our group vs. 16.5% (14.3 against 12.2% among asymptomatic people). Although it is not possible to make conclusions from this comparison, the possibility that VGC treatment gave us good results on this specific follow-up must be considered.

The language evaluation was normal in all but six patients, three of whom were symptomatic at birth with CNS involvement. A significant difference emerged between those with and without language disorders (*p* = 0.042 at the Mann–Whitney test) in relation to the number of VGC cycles needed to achieve persistent negative viremia. Importantly, total non-negligible lower scores in semantic fluency tests were found. Examining only the asymptomatic, seven patients out of nine showed low scores.

Focusing attention on the results of the behavioral questionnaire (CBCL), a quarter of patients has a trait of weak psychic structure on the internalizing side, with a tendency toward anxiety and social withdrawal. None of the patients was diagnosed with autism spectrum disorder (as in 3% of the Korndewal's group, not treated with VGC) (9). The behavioral and emotional spheres are obviously multifactorial. Furthermore, the sequelae of cCMV in the auditory and ophthalmologic areas and the consequent possible need to wear hearing aids and/or glasses and performing rehabilitative therapies are sources of stress for the child and embarrassment with peers. A recent study from Switzerland confirmed the abnormal neuro-development of patients with cCMV (25).

The outcome of post-treatment audiological evaluations did not show the onset of SNHL in normal hearing patients or its deterioration in those in whom it was already present at birth, except in one case. This observation suggests that the control of viral replication in the first year of life, through the administration of VGC, may limit the direct or immune-mediated damage caused by CMV. Only one child had post-treatment worsening of auditory function. Symptomatic at birth (thrombocytopenia, neutropenia, and petechiae), he developed hearing loss during the 1st year of life. At 15 months of age, hearing function worsened and the use of a right prosthesis was implemented in the second year of life. Following a new deterioration at the age of 3 he began using a prosthesis also for the left ear. On this occasion, we administered VGC for 6 months, after which the hearing thresholds were confirmed stable. Our choice was based on the hypothesis that the observed damage could be the result of long lasting inflammation and, therefore, the control of viral replication could influence the audiological outcome, as hypothesized by the study of Kimberlin et al. (14). They compared VGC treatment (six weeks vs. six months) in patients with symptomatic cCMV infection. This study demonstrated comparable efficacy in terms of 6-month audiological outcome in the two groups. Instead, the prolonged treatment demonstrated a statistically significant superiority for the same outcome at 12 and 24 months, with a maintenance of the hearing benefit. In addition, a statistically significant superiority of the 6-month treatment in linguistic and communicative-behavioral development was reported (14).

Our data confirm what is already known in literature: symptomatic cCMV is a risk factor for the presence of damage to the auditory function (*p* = 0.012). Comparing with the Korndewal's group of untreated patients, the prevalence of hearing loss is proportionally greater in our sample. However, the picture of the audiological status of the patients examined by us after years of treatment reflects in almost all cases what has already been present at birth, or pre-treatment (9).

Regarding the ophthalmological follow-up, the absence of development of chorioretinitis, possible outcome of the infection, is also a positive finding. The results are undoubtedly encouraging, especially when compared with a recent publication of ophthalmological interest in which the development of retinal damage was recorded in 7.8% of symptomatic and in 3.7% of asymptomatic untreated patients (26).

Finally, some considerations are important regarding the trimester of maternal infection. As already described, cCMV infection is more severe in newborns born to pregnant women with first trimester infection. However, the long-term outcome shows a varied distribution of falls in specific scales of cognitive development, language and behavioral sphere. An extremely important finding is that neither a group of patients is free from problems nor it is characterized by a recurrence of specific sequelae.

Our study has some limitations: first, we describe a small sample size; secondly, we do not have a control group of not treated newborns with cCMV to compare with those treated. Despite these limits, our study clearly shows that both symptomatic and asymptomatic newborns with cCMV infection develop long-term sequelae, particularly in the behavioral and communicative areas, no matter the trimester of maternal infection. Importantly, our study also shows a possible association between the entity of viral replication and future sequelae, suggesting that controlling it with antiviral treatment appears a reasonable strategy. Finally, although no comparison with untreated cCMV has been done, our series of treated patients shows a better neuro-cognitive and audiological long-term outcome compared to available data from literature about untreated patients.

New studies evaluating more patients with symptomatic and asymptomatic CCMV and including a randomization of different treatment strategies are needed to better define the best way to manage this increasingly common and characterized condition.

## Data Availability Statement

The datasets generated for this study are available on request to the corresponding author.

## Ethics Statement

The Ethics Committee of Catholic University of Sacred Heart/Fondazione Policlinico Universitario Agostino Gemelli IRCCS approved this study.

## Author Contributions

AT, VF, DP, and PV contributed conception and design of the study. GS, DC, DR, GC, FM, AB, RL, and RS collected the data. AT and VF organized the database. CD and AA performed the statistical analysis. AT wrote the first draft of the manuscript. DB and DP wrote sections of the manuscript. All authors contributed to manuscript revision, read, and approved the submitted version.

## Conflict of Interest

The authors declare that the research was conducted in the absence of any commercial or financial relationships that could be construed as a potential conflict of interest.
